# Transitioning to peritoneal dialysis: it does not matter where you come from

**DOI:** 10.1590/2175-8239-JBN-2023-0139en

**Published:** 2024-05-06

**Authors:** Diogo Francisco, Andreia Carnevale, Gonçalo Ávila, Ana Rita Calça, Patrícia Matias, Patrícia Branco

**Affiliations:** 1 Centro Hospitalar Lisboa Ocidental, Serviço de Nefrologia, Lisboa, Portugal.

**Keywords:** Kidney Failure, Chronic, Renal Dialysis, Kidney Transplantation, Peritoneal Dialysis, Renal Replacement Therapy

## Abstract

**Introduction:**

Patients with end-stage renal disease (ESRD) frequently change renal replacement (RRT) therapy modality due to medical or social reasons. We aimed to evaluate the outcomes of patients under peritoneal dialysis (PD) according to the preceding RRT modality.

**Methods:**

We conducted a retrospective observational single-center study in prevalent PD patients from January 1, 2010, to December 31, 2017, who were followed for 60 months or until they dropped out of PD. Patients were divided into three groups according to the preceding RRT: prior hemodialysis (HD), failed kidney transplant (KT), and PD-first.

**Results:**

Among 152 patients, 115 were PD-first, 22 transitioned from HD, and 15 from a failing KT. There was a tendency for ultrafiltration failure to occur more in patients transitioning from HD (27.3% vs. 9.6% vs. 6.7%, p = 0.07). Residual renal function was better preserved in the group with no prior RRT (p < 0.001). A tendency towards a higher annual rate of peritonitis was observed in the prior KT group (0.70 peritonitis/year per patient vs. 0.10 vs. 0.21, p = 0.065). Thirteen patients (8.6%) had a major cardiovascular event, 5 of those had been transferred from a failing KT (p = 0.004). There were no differences between PD-first, prior KT, and prior HD in terms of death and technique survival (p = 0.195 and p = 0.917, respectively) and PD efficacy was adequate in all groups.

**Conclusions:**

PD is a suitable option for ESRD patients regardless of the previous RRT and should be offered to patients according to their clinical and social status and preferences.

## Introduction

Chronic kidney disease (CKD) is largely a preventable and treatable disease that is estimated to affect 9.1% of the world population^1^. An estimated glomerular filtration rate (eGFR) <15 mL/min/1.73 m^2^ defines end-stage renal disease (ESRD)^2^, which can be treated with either dialysis (hemodialysis or peritoneal dialysis), kidney transplantation, or a conservative approach.

In 2010, 2.618 million people received renal replacement therapy (RRT) worldwide. However, it is estimated that between 4.902 and 9.701 million people need RRT^3^. Disparities in CKD-associated mortality reveal regional asymmetries in access to dialysis. It is estimated that 1-2 million people globally have died prematurely due to lack of access to RRT in 2017^1,4^.

Preemptive kidney transplantation (KT) with a living donor is the preferred treatment for transplant-eligible CKD patients^5^. Despite many advances in immunosuppression safety and efficacy that allowed for the prolongation of graft survival, many patients must return to dialysis after a period with a functioning graft. According to the United States Renal Data System (USRDS), adjusted 10-year graft survival of living donor and deceased donor was 65.5% and 49.5%, respectively. Patients who were treated with peritoneal dialysis (PD) after graft failure were more likely to receive a subsequent kidney transplant than to die over the ensuing three years; the opposite was true for patients who were treated with hemodialysis (HD)^6^.

There is no preferred modality when starting dialysis after a failed renal graft, as both PD and HD seem suitable options^7–9^. Optimal immunosuppression management in patients starting dialysis with a failing KT is still uncertain, as data in this field is scarce. A failed graft represents a chronic inflammatory stimulus that might negatively affect nutritional status and cardiovascular risk, and preservation of residual graft function might positively impact PD outcomes, as it happens with native kidneys. Clinicians should weigh the risks and benefits of withdrawing the immunosuppressive therapy. In case of maintenance of immunosuppression, anti-proliferative drugs should be discontinued first. Calcineurin-inhibitors (CNI) should be tapered over several weeks and glucocorticoids over several months aiming at residual renal function (RRF) preservation and avoiding renal graft rejection^7,10^.

Although costly^11^, HD is currently the most common RRT offered to patients with ESRD, despite current recommendations that HD should be the lowest priority in a CKD treatment program, after prevention of CKD progression, conservative treatment when suitable, KT, and PD^4,12^.

The association of PD with better clinical and patient-reported outcomes is well established. These benefits include better preservation of RRF, improved quality of life, preservation of vascular territories for subsequent vascular access construction, and better subsequent KT outcomes^13^. PD has several features that could make it appealing for preferential RRT in both low-to-middle-income countries and high-income countries. It is technically simpler and more cost-effective, requires a lower nurse-to-patient ratio, is more feasible in rural and remote regions, provides greater equity in resource-limited settings, and may improve survival in the first years^13^. Despite its potential advantages, only 8–12% of ESRD worldwide are under PD^14,15^. Multiple factors contribute to regional differences in RRT modalities, including government dialysis policies and financing, healthcare system and facility factors, and patient comorbidities and suitability, as well as industry factors^16^.

Patients under chronic RRT frequently change modality due to medical or social reasons. *Transition* is the term for the process that should include preparation and adaptation periods to the new reality.

Peritoneal ultrafiltration and diffusive capacity often decreases with time. A unified definition of PD technique failure has been proposed that includes a composite endpoint of transfer to HD or death^13^. Peritonitis and PD-related infections are the major causes for technique failure, which is associated with higher mortality^13^. RRF is a surrogate marker of PD strongly associated with improved patient and technique survival^13^.

Patient-centered innovations that support more efficient kidney care and improve patient outcomes are increasingly demanded^17^. In this study, we aimed to evaluate the outcomes of patients under PD according to the preceding RRT modality.

## Methods

This was a retrospective observational single-center study approved by our hospital’s Ethics Committee. We evaluated all ESRD patients who started PD at our unit from January 1, 2010, to December 31, 2017. Incident patients with less than 3 months under PD were excluded. Patients were followed for 60 months or until they dropped out of PD. Patients under continuous ambulatory peritoneal dialysis (CAPD) and automated peritoneal dialysis (APD) were included.

We selected three cohorts of patients. The PD-first cohort included patients with no prior RRT; the previous HD group included patients who transitioned directly from HD; and the prior KT group included patients who transitioned to PD directly from a failing KT.

Charlson comorbidity index is a validated prognosis tool used to evaluate disease burden and 10-year mortality rate^18^. We calculated the comorbidity burden according to the Charlson score.

Initial Peritoneal Equilibration Test (PET) was performed 3–6 months after PD initiation and then every 6–12 months according to clinical needs. A modified protocol using 3.86%/4.25% glucose was used. Blood samples were collected at the time of infusion and 2 hours after infusion. Peritoneal fluid samples are drawn at 0, 2, and 4 hours; peritoneal fluid is completely drained 4 hours after infusion. A 24-hour urine collection and a 24-hour peritoneal effluent were analyzed. PET results included weekly Kt/V, diuresis and eGFR, creatinine D/P ratio, and nutritional evaluation with the normalized protein catabolic rate (nPCR)^19^. Ultrafiltration (UF) capacity was also evaluated and UF failure was defined as net UF < 400 mL^20^.

Technique failure was evaluated through a composite endpoint of death or HD transfer^13^, and technique survival was defined as the interval between PD start and technique failure. At our center, a strategy to reduce glucose burden in diabetic patients was implemented with the use of glucose-free peritoneal dialysis solutions, such as icodextrin or amino acid-based solutions^21^.

Immunosuppression protocols for patients who started PD from a failing KT included immediate withdrawal of anti-proliferative drugs and progressive tapering of CNI. Glucocorticoids were maintained until there is no residual diuresis. Patients who were previously on mammalian target of rapamycin (mTOR) inhibitors were switched to CNI before PD catheter placement to avoid healing delay.

### Statistical Analysis

Continuous variables are presented as mean and standard deviation for normally distributed variables or as median and interquartile range (IQR) for non-normally distributed variables, and categorical variables were reported as frequencies or percentages. Inferential statistical analysis included the Kruskal-Wallis test to compare continuous variables with non-normal distribution, one-way ANOVA to compare continuous variables with normal distribution, and chi-square test or Fisher exact test to compare categorical variables.

Kaplan-Meyer survival curves were performed. Multivariable Cox regression analysis was used to analyze clinical variables independently associated with PD failure (death or HD transfer) during the follow-up period. Variables with p < 0.20 or selected at the discretion of the investigator in univariable analysis were included in the multivariable model. Differences with a p < 0.05 were considered significant. Statistical analysis was performed using the SPSS program v. 22.0 (SPSS Inc., Chicago, IL, USA).

## Results

**Figure 1:**
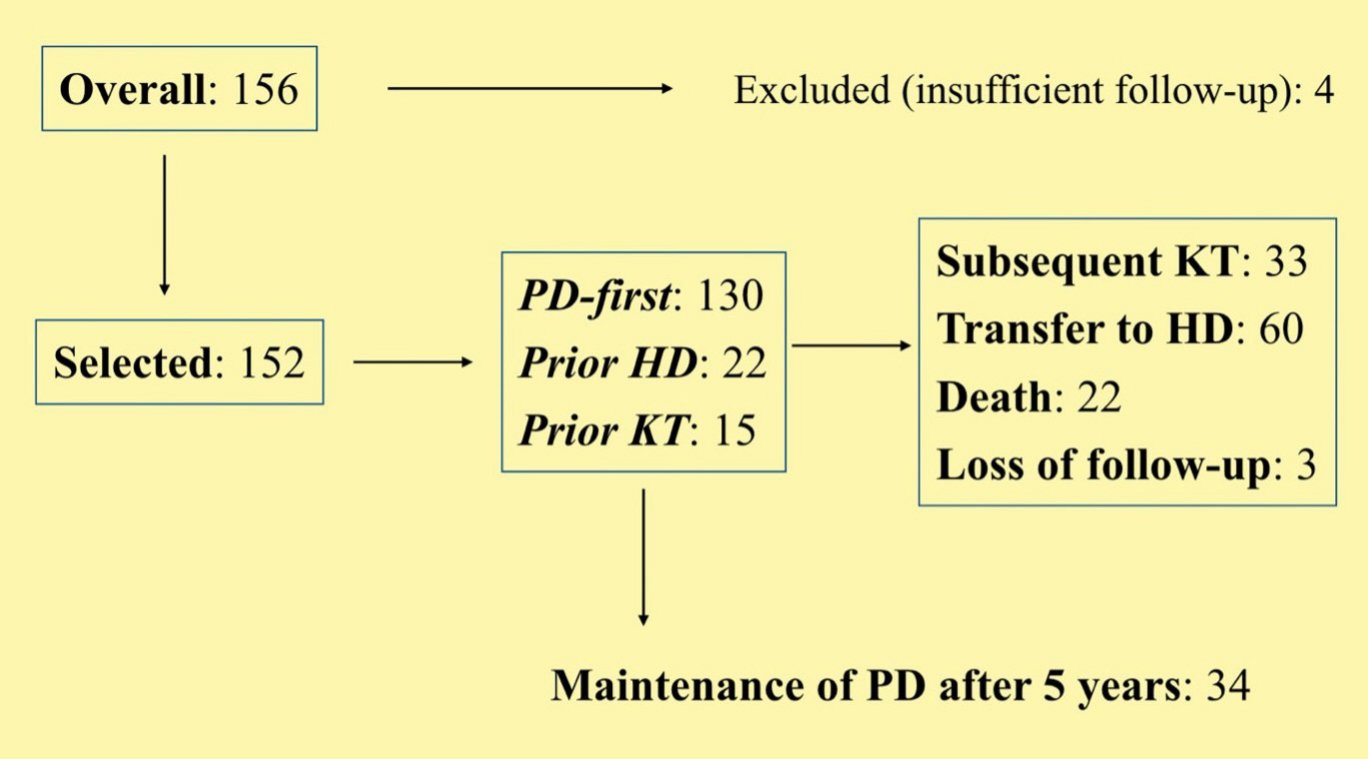
Flow chart of patient selection and follow-up. HD – Hemodialysis; KT – Kidney Transplantation; PD – Peritoneal Dialysis.

There were 156 patients starting PD in the studied period, of which 4 were excluded due to insufficient follow-up time. Among 152 patients included in our study, 115 were PD-first, 22 transitioned from HD, and 15 transitioned from a failing KT (Figure 1). The median follow-up time was 45.72 months (IQR 37.44). The population included 61.8% male patients, with a mean age of 51.1 ± 16.5 years at PD initiation. More than one-third (36.8%) had diabetes, and median Charlson score was 4.0 (IQR 4.0) with 51.3% of the patients having a score of 3–6. Almost one-third (29.6%) had no relevant comorbidities other than ESRD (Charlson score of 2). Patients with a Charlson score > 2 had a higher rate of technique failure (60.7% *vs*. 37.8%, p = 0.012). There was no statistical difference among groups regarding overall mortality and between patients with a Charlson score > 2 (Table 1).

**Table 1:** Patient demographics, comorbidity, and clinical events stratified for previous RRT

Variable	Total	Previous RRT			p
		KT (n = 15)	HD (n = 22)	PD-first (n = 115)	
Age at RRT start (y), mean ± SD	51.1 ± 16.5	30.0 ± 14.6	51.9 ± 13.5	53.8 ± 15.3	**<0.001**
RRT vintage (y), median (IQR)	3.8 (3.1)	15.2 (8.0)	4.6 (2.8)	4.3 (2.7)	**<0.001**
Age at PD start (y), mean ± SD	52.6 ± 15.2	41.0 ± 16.5	52.3 ± 15.4	53.0 ± 14.5	0.030
Time under PD (y), median (IQR)	3.81 (3.12)	2.78 (3.20)	3.69 (3.19)	4.3 (2.6)	0.230
Male, n (%)	94 (61.8)	6 (40.0)	12 (54.5)	76 (66.1)	0.110
Diabetes *mellitus*, n (%)	56 (36.8)	2 (13.3)	8 (36.4)	46 (40.0)	0.131
Hypertension, n (%)	99 (65.1)	9 (60.0)	11 (50.0)	79 (68.7)	0.219
Charlson score, median (IQR)	4.0 (4.0)	2.0 (4.0)	4.0 (4.0)	4.0 (4.0)	0.151
Charlson score, n (%)	–	–	–	–	0.231
2	45 (29.6)	8 (53.3)	5 (22.7)	32 (27.8)	–
3–6	78 (51.3)	6 (40.0)	14 (63.6)	58 (50.4)	–
Above 6	29 (19.1)	1 (6.7)	3 (13.6)	25 (21.7)	–
Hospital admission annual rate, median (IQR)	0.60 (1.34)	1.08 (0.66)	0.38 (1.33)	0.40 (0.96)	**0.011**
Annual peritonitis rate, median (IQR)	0.21 (0.66)	0.70 (0.75)	0.10 (0.91)	0.21 (0.55)	0.065
IOS, n (%)	64 (42.1)	8 (53.3)	7 (31.8)	49 (42.6)	0.418
Tunnel infection, n (%)	28 (18.4)	4 (26.7)	3 (13.6)	21 (18.3)	0.631
MACE, n (%)	13 (8.6)	5 (33.3)	–	8 (7.0)	**0.004**
Temporary drop-out, n (%)	63 (41.4)	9 (60.0)	8 (36.4)	46 (40.0)	0.292
Drop out, n (%)	–	–	–	–	0.494
PD maintenance	34 (22.4)	3 (20.0)	4 (18.2)	27 (23.5)	–
Hemodialysis transfer	60 (39.5)	6 (40.0)	5 (22.7)	49 (42.6)	–
KT	33 (21.7)	4 (26.7)	7 (31.8)	22 (19.1)	–
Loss of follow up	3 (2.0)	–	–	3 (2.6)	–
Death	22 (14.5)	2 (13.3)	6 (27.3)	14 (12.2)	0.195
Technique failure, n (%)	82 (53.9)	8 (53.3)	11 (50.0)	63 (54.8)	0.917

Sixty-two patients (40.8%) did not have any peritonitis event during the follow-up, 57.9% had no exit site infection, and 81.6% had no tunnel infection. There were no significant differences among groups in the incidence of tunnel and exit site infections, and a tendency without statistical significance towards a higher annual peritonitis rate was observed in the previous KT group (median of 0.70 peritonitis/year per patient *vs*. 0.10 in HD and 0.21 in PD, p = 0.065). Thirteen patients (8.6%) had a major cardiovascular event (MACE), of which 5 had been transferred from a failing KT (33.0% *vs*. 7.0% *vs*. 0%, p = 0.004). Around one-third of the patients (n = 48, 31.6%) had no hospital admission during the follow-up. However, the annual hospital admission rate in the prior KT group was 1.08 admission/year per patient, higher than in the other groups (0.38 in HD and 0.40 in PD-first, p = 0.011). The main reasons for hospital admission in the previous KT group were PD-related infections (n = 19; 35.8%), cardiovascular events (n = 13; 24.5%), non-PD-related infections (n = 6; 11.3%), and non-infectious problems related to PD (n = 6; 11.3%). There were no differences between APD and CAPD patients regarding the annual hospital admission rate or peritonitis incidence.

Patients who transitioned from HD to PD had a mean age of 51.9 ± 13.5 years and a median HD vintage of 0.93 years (IQR 1.77) years. The main reason for the transition to PD was the patient’s option (n = 14; 63.6%), followed by problems with vascular access for HD (n = 7; 31.8%), and intolerance to ultrafiltration in HD (n = 1; 4.5%).

Patients in the prior KT group had received a kidney graft at a mean age of 33.0 ± 14.7 years, and the duration of the KT had a median of 9.8 years (IQR 5.0). After transitioning to PD, non-glucocorticoid immunosuppression was maintained for a median of 181.5 days (IQR 176.0). Glucocorticoids were maintained for a longer time, while the patient still had RRF. This group was considerably younger when they started RRT (mean of 30.0 *vs*. 51.9 in HD *vs*. 53.8 in PD-first, p < 0.001) and when they started PD (mean of 41.0 *vs*. 52.3 in HD *vs*. 53.0 in PD-first, p = 0.030). Their vintage on ESRD is also considerably longer (median of 15.2 years *vs*. 4.6 in HD and 4.3 in PD-first, p < 0.001).

Sixteen patients did not perform any PET, and 18 did perform a first PET but did not perform a second exam due to early PD withdrawal. Average dialysis efficacy was adequate (Kt/V>1.7) in all groups, both at PD start and at the end of follow-up. Median diuresis at PD beginning was 1525 mL (IQR 1400), which corresponded to a median GFR of 6.3 mL/min/1.73 m^2^ (IQR 5.6). Median annual rate of diuresis reduction was 305 mL (IQR 703).

Patients under CAPD (114) and APD (38) were included in the study. Patients under CAPD were older (54.9 *vs*. 46.1, p = 0.002). There were no differences in Kt/V at PD start (p = 0.591) or at the end of follow-up, as well as in GFR at the end of follow-up. Patients under CAPD had a higher comorbidity index compared with patients under APD (Charlson index > 2 of 77.0% *vs*. 52.6%, p = 0.007).

At the beginning of PD, there were 17 patients already anuric. Three of the patients were from the prior KT group (20% of all prior KT*)*, 4 from prior HD (18.2% of all prior HD), and 10 from PD-first (8.7% of all PD-first). Regarding RRF, diuresis at PD start was lowest in the prior KT group and highest in PD-first group (750 mL/day *vs*. 1300 mL/day *vs*. 1825 mL/day, p < 0.001), as was by the end of follow-up (0 *vs*. 300 *vs*. 1250 mL, p < 0.001). GFR followed the same pattern (at PD start 2.2 *vs*. 6.3 *vs*. 7.0 mL/min, p < 0.001; at end of follow-up 0.0 *vs*. 1.5 *vs*. 3.8 mL/min, p < 0.001). The annual percentages of diuresis and measured GFR lost during follow-up were higher in the group of patients transitioning from a failing KT (annual percentage of diuresis lost 0.59 in prior KT *vs*. 0.13 *vs*. 0.13%, p = 0.044; and annual percentage of measured GFR loss of 0.60 *vs*. 0.16 *vs*. 0.21%, p = 0.042).

Eighteen patients (11.8%) developed UF failure during the study. UF failure showed a tendency – non-significant – towards lower incidence in the prior KT group (6.7%) and higher in the prior HD group (27.3%, p = 0.070). Although most patients were intermediate solute transporters by the end of follow-up, 3.3% were slow solute transporters and a higher percentage of patients (8.6%) were fast solute transporters. Overall adequate nutritional status was also achieved, although a reduction from an initial nPCR of 1.02 ± 0.28 to 0.89 ± 0.25 was observed. No statistically significant difference was identified between groups (Table 2).

**Table 2:** Dialysis efficacy stratified for previous RRT

Variable	Total	Previous RRT			p
		KT (n = 15)	HD (n = 20)	PD-first (n = 101)	
Creatinine D/P at PD start, mean ± SD	0.69 ± 0.10	0.72 ± 0.03	0.67 ± 0.11	0.69 ± 0.11	0.492
Creatinine D/P at end of follow up, mean ± SD	0.68 ± 0.11	0.71 ± 0.10	0.66 ± 0.12	0.68 ± 0.10	0.487
Peritoneal transport at end of follow up	–	–	–	–	0.977
Slow	5 (3,3)	–	1 (4.5)	4 (3.5)	–
Intermediate-slow	43 (28.3)	4 (26.7)	7 (31.8)	32 (27.8)	–
Intermediate-fast	57 (37.5)	6 (40.0)	7 (31.8)	44 (38.3)	–
Fast	13 (8.6)	1 (6.7)	1 (4.5)	11 (9.6)	–
Kt/v (at PD start), mean ± SD	2.59 ± 0.81	2.24 ± 0.42	2.51 ± 0.60	2.69 ± 0.87	0.052
Kt/v (at end of follow up), mean ± SD	2.08 ± 0.62	1.79 ± 0.22	2.01 ± 0.76	2.12 ± 0.62	0.162
Annual rate of Kt/v change, mean ± SD	0.25 ± 0.50	0.30 ± 0.36	0.17 ± 0.48	0.27 ± 0.52	0.716
Diuresis at PD start (mL), median (IQR)	1525 (1400)	750 (1260)	1300 (1100)	1825 (1300)	**<0.001**
Diuresis at end of follow up (mL), median (IQR)	1000 (1300)	0 (200)	300 (1465)	1250 (1175)	**<0.001**
Annual variation of residual diuresis (%), mean ± SD	0.17 ± 0.55	0.59 ± 0.41	0.13 ± 0.88	0.13 ± 0.49	**0.044**
GFR at PD start (mL/min/1.73 m^2^), median (IQR)	6.3 (5.6)	2.2 (5.1)	6.3 (7.5)	7.0 (5.4)	**<0.001**
GFR at end of follow up (mL/min/1.73 m^2^), median (IQR)	2.6 (4.7)	0.0 (0.6)	1.5 (3.6)	3.8 (4.5)	**<0.001**
Annual variation in GFR (%), mean ± SD	0.24 ± 0.48	0.60 ± 0.39	0.16 ± 0.56	0.21 ± 0.47	**0.042**
UF failure, n (%)	18 (11.8)	1 (6.7)	6 (27.3)	11 (9.6)	0.070
nPCR at PD start (g/kg/day), mean ± SD	1.02 ± 0.28	0.96 ± 0.17	0.95 ± 0.28	1.04 ± 0.28	0.198
nPCR at end of follow up (g/kg/day), mean ± SD	0.89 ± 0.25	0.91 ± 0.19	0.81 ± 0.30	0.90 ± 0.25	0.382
Annual loss of nPCR (g/kg/day), mean ± SD	0.69 ± 0.21	0.02 ± 0.14	0.04 ± 0.14	0.08 ± 0.23	0.604

Sixty-three patients (41.4%) were temporarily out of PD due to infections or abdominal surgeries and resumed PD after a short period. On the other hand, definitive PD withdrawal before 60 months of follow-up occurred in 77.6% of patients. A total of 33 patients (21.7%) received a KT during the follow-up.

Half of all patients (n = 82; 53.9%) developed technique failure (death or HD transfer) after a median of 2.32 years (IQR 2.53) of PD. Overall, the majority of patients were transferred to HD (39.5%) and the main reasons were UF failure or loss of dialysis efficacy (n = 21; 35%), PD-related infectious complications (n = 20; 33%), PD-catheter-related mechanical problems (n = 7; 12%), loss of autonomy to perform PD and absence of a helper (n = 7; 12%), and patient option (n = 5; 8%).

Despite an overall mortality rate of 14.5%, there was a statistically non-significant tendency towards higher mortality rate in patients transitioning directly from HD (27.3% *vs*. 13.3% *vs*. 12.2%, p = 0.195). In the group of patients who transitioned directly from HD, the main reasons for PD withdrawal was KT (31.8%) followed by death (27.3%).

**Figure 2:**
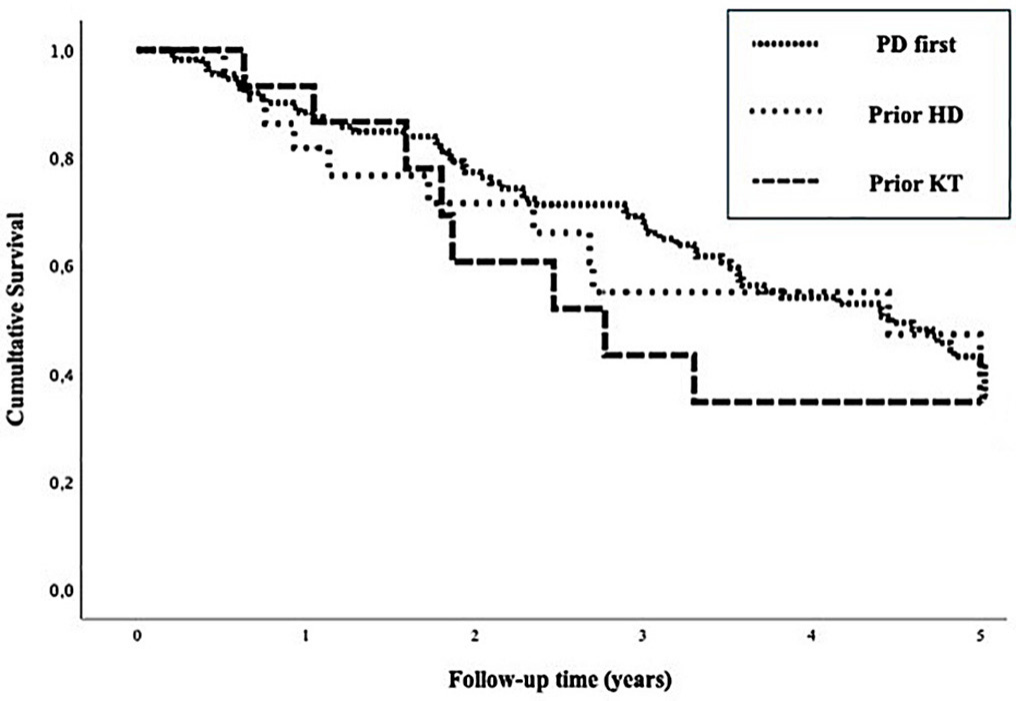
Technique survival curves of each group for a composite endpoint of death or hemodialysis transfer, p = 0.612.

In the multivariable analysis, neither drop-out from PD (p = 0.494) nor technique survival (p = 0.917, log rank = 0.612, Figure 2) were different among groups. The annual hospital admission rate decreased both technique survival and patient survival (HR 1.536, 95%CI 1.102–2.140, p = 0.011 and HR 1.797, 95%CI 1.100–2.650, p = 0.017, respectively). Diabetes was an independent risk factor for death and for technique failure (HR 2.694, 95CI% 1.102–6.586, p = 0.030 and HR 1.696, 95%CI 1.047–2.747, p = 0.032) (Tables 3 and 4). In addition, neither type of PD (CAPD *vs.* APD) nor type of transition (PD-first *vs*. prior HD *vs*. failing KT) influenced overall mortality or technique survival.

**Table 3:** Cox regression for death

Variable	HR	95% CI	p
Diabetes mellitus	2.694	1.102–6.586	0.030
Annual hospital admission rate	1.797	1.100–2.650	0.017

Variables in the model: age at PD start, diabetes mellitus, hospital admission, and peritonitis annual rate.

**Table 4:** Cox regression for technique failure

Variable	HR	95%CI	p
Diabetes mellitus	1.696	1.047–2.747	0.032
Annual hospital admission rate	1.536	1.102–2.140	0.011

Technique failure is a composite endpoint of death and transfer to hemodialysis. Variables in the model: urine output at PD start, diabetes mellitus, hospital admission and peritonitis annual rate.

## Discussion

The transition between RRT techniques can be a stressful event for ESRD patients, who have to adapt to new challenges and daily routines. We hypothesized that the RRT technique before PD could influence PD outcomes. Among 152 patients, 115 were PD-first, 22 transitioned from HD, and 15 were from a failing KT. Patients transitioning from a failing KT presented a tendency towards a higher annual rate of peritonitis and hospital admissions. Urinary output by the end of follow-up was lower in patients transitioning from a failing KT or from HD. Patients transitioning from HD presented a tendency towards a higher prevalence of UF failure, which was not statistically significant. The presence of diabetes and hospital admissions were associated with a higher probability of death or HD transfer.

The prevalence of diabetes among PD patients was 36.8%, which is lower than in the general PD population in the US (59.9%)^14^. These differences do not seem to reflect differences in diabetes prevalence in both countries, which are similar (around 10%)^22,23^. Both patient and technique survival were influenced by the presence of diabetes. Death probability during follow-up more than doubled in diabetic patients (HR 2.694, 95%CI 1.102–6.586, p = 0.030). Diabetes is an important cardiovascular risk factor, and cardiovascular disease is the main cause of death in ESRD patients^24^. Although the results did not show statistical significance, diabetic patients had a higher incidence of major cardiovascular events during follow-up (14.3% *vs*. 5.2%, p = 0.072).

Technique failure probability – a composite endpoint of death and transfer to HD – was 69.9% higher in diabetic patients. Despite the implementation of glucose-sparing regimens of PD at our unit^21^, peritoneal exposure to glucose in diabetic patients under PD promotes greater peritoneal fibrosis which could lead to technique failure. Patient survival among diabetic patients under PD and HD is comparable. Cotovio et al.^25^ found that diabetes was an independent risk factor for death but not for technique failure. In their analysis, the peritonitis rate was similar between nondiabetic and diabetic patients, but the hospitalization rate was higher among diabetics^25,26,27^.

Educational level appears to be associated with the risk of peritonitis regardless of economic status^28,29^. In our population, the overall annual peritonitis rate is below the guidelines recommended by the International Society of Peritoneal Dialysis (ISPD) (0.21 *vs*. 0.40)^30^. However, patients starting PD from a failing KT had a higher rate of peritonitis and this was the main reason for hospital admission in this cohort. Interestingly, the peritonitis rate had no influence on the probability of death or technique failure. On the other hand, the annual hospital admission rate was influenced by both variables. Death probability increased by 79.7% and technique failure increased by 53.6% for each point increase in the annual admission rate. Hospital admission rate reflects PD and ESRD complications, such as infectious and cardiovascular events, which have an impact in PD outcomes.

In general, dialysis efficacy (weekly Kt/V) was adequate, regardless of prior RRT, diabetes status, or PD modality (APD *vs*. CAPD). All groups progressively lost diuresis and residual kidney function. However, PD-first patients had significantly higher daily urinary volume than other groups by the end of follow-up. Loss of urinary volume in prior KT group might reflect the accelerated loss of residual renal function in KT with the progressive withdrawal of immunosuppression and possible chronic graft rejection. Patients who transitioned from HD also developed lower urinary output at the end of follow-up. PD appears to preserve residual renal function better than HD^31,32,33^.

An additional problem was observed among patients who transitioned from HD – a tendency towards a higher incidence of UF failure. There is no clear explanation for this unexpected finding. It was the group with the lowest incidence of infections – an important risk factor for UF insufficiency. The incidence of diabetes was not greater than that in the PD-first group, which has a much lower UF failure rate. The prior KT group – which had at least one major abdominal surgery and the lowest incidence of diabetes, despite years of immunosuppression – had the lowest incidence of UF insufficiency. Again, we might speculate whether the inflammatory stimulation triggered by the extracorporeal circuit could influence both residual renal function and peritoneal membrane function. The trend towards a higher prevalence of UF failure and lower urinary output in patients transitioning from HD could suggest that volume status control in this group is more challenging.

Our findings were compared with a Spanish cohort (n = 906) of PD patients who had no prior RRT or transitioned from a failing KT. We found that our cohort was younger (51.1 *vs*. 54.8, p = 0.008) and had a lower comorbidity index (4.0 *vs*. 5.1, p < 0.001). Our cohort presented a higher incidence of diabetes (36.8% *vs*. 24.0% p < 0.001), higher mortality (14.5 *vs*. 9.7%, p = 0.084), and higher HD transfer rate (39.5% *vs*. 17.0%, p < 0.001)^8^. Better outcomes in the Spanish cohort might be associated with a much lower diabetes prevalence.

Prior HD patients were under HD for a median of less than one year before transfer to PD. The first reason to PD withdrawal was KT (31.8%) – more than any other group. This might reflect the phenomenon of *crashlanding* in HD before deciding on a preference for PD as a bridge to KT. Interestingly, patients who started PD from HD were also the group with the highest mortality rate. As discussed previously, the tendency for higher rate of UF failure and lower urinary output at the end of follow-up might have affected the outcomes.

During the 8 years of follow-up, only 9.9% of patients starting PD came from KT. The prior KT group had a significantly higher annual rate of peritonitis (0.70 episodes/year per patient) when compared to other PD patients, which is above what is recommended by ISPD^30^. Major cardiovascular events were also more common in this group (33.0% *vs*. 7.0% *vs*. 0, p = 0.004). Probably, accelerated loss of diuresis associated with long-term uremia and chronic immunosuppression favors cardiovascular disease development and infections. However, no differences were found in dialysis efficacy, and prior KT patients had the same rates of technique failure and death as the overall population. Therefore, despite a higher risk of infection and cardiovascular disease, PD remains a suitable option for patients with a failing KT. Control of cardiovascular risk factors should be intensified with further advice on lifestyle modifications, such as smoking cessation, body weight control, adoption of a healthy diet, and exercise^34^. Cardiovascular risk factors such as diabetes and hypertension – but also mineral-bone disease – should be thoroughly controlled. Intensification of learning sessions with specialized nurses should be regularly offered to these patients, to minimize technical errors that could lead to infections^30,35^.

Our study is limited by being unicentric, retrospective, and observational, and had an asymmetry in the size of the groups studied. Particularly, there was a lower number of patients in the prior KT and HD groups. Also, the limited number of patients transitioning from a failing KT did not allow an analysis of different immunosuppression withdrawal strategies after PD initiation. However, this study enrolled all patients from a unit without pre-selection and had a long follow-up. To the best of our knowledge, this is one of the first studies that simultaneously compared 3 cohorts of PD patients according to their previous RRT, namely HD *vs*. KT *vs*. PD-first.

## Conclusions

We evaluated a large population of prevalent patients under PD according to their previous RRT with a long follow-up. Patients transitioning from HD or from a prior KT appear to have lower urinary output by the end of the follow-up. A strong tendency for higher rate of UF failure in patients transitioning from HD might anticipate difficulties in volume status control in this group. Patients transitioning from a failing KT had a higher rate of both peritonitis and hospital admissions. Presence of diabetes and hospital admission were associated with a higher probability of death or HD transfer. PD efficacy indicators were adequate in all of the studied groups.

Despite the previously described differences and according to the literature, PD appears to be a valid choice of chronic RRT after a failed KT or HD and should be offered to patients according to their clinical and social status and preferences.
