# Supplementary material for: Transitioning to peritoneal dialysis: it does not matter where you come from
Source: J Bras Nefrol. 2024 May 6;46(3):e20230139. doi: 10.1590/2175-8239-JBN-2023-0139en (PMC11287956; doi:10.1590/2175-8239-JBN-2023-0139en)
Supplement: Translation

# Translation: Transição para diálise peritoneal: não importa de onde

**DOI:** 10.1590/2175-8239-JBN-2023-0139pt

**Authors:** Diogo Francisco, Andreia Carnevale, Gonçalo Ávila, Ana Rita Calça, Patrícia Matias, Patrícia Branco

**Affiliations:** 1 Centro Hospitalar Lisboa Ocidental, Serviço de Nefrologia, Lisboa, Portugal.

**Keywords:** Falência Renal Crônica, Diálise Renal, Transplante de Rim, Diálise Peritoneal, Terapia de Substituição Renal

## Abstract

**Introdução:**

Pacientes com doença renal em estágio terminal (DRET) frequentemente mudam de modalidade de terapia renal substitutiva (TRS) por razões médicas ou sociais. Nosso objetivo foi avaliar desfechos de pacientes em diálise peritoneal (DP) segundo a modalidade anterior de TRS.

**Métodos:**

Realizamos estudo retrospectivo observacional unicêntrico, em pacientes prevalentes em DP, de 1º de janeiro de 2010 a 31 de dezembro de 2017, acompanhados por 60 meses ou até saírem de DP. Pacientes foram divididos em três grupos de acordo com a TRS anterior: hemodiálise prévia (HD), transplante renal malsucedido (TR) e DP como primeira opção (PD-first).

**Resultados:**

Entre 152 pacientes, 115 eram PD-first, 22 transitaram da HD e 15 de TR malsucedido. Houve tendência à maior ocorrência de falência de ultrafiltração em pacientes em transição da HD (27,3% vs. 9,6% vs. 6,7%; p = 0,07). A função renal residual foi melhor preservada no grupo sem TRS prévia (p < 0,001). Observou-se tendência à maior taxa anual de peritonite no grupo TR prévio (0,70 peritonite/ano por paciente vs. 0,10 vs. 0,21; p = 0,065). Treze pacientes (8,6%) tiveram um evento cardiovascular maior, cinco dos quais haviam sido transferidos de um TR malsucedido (p = 0,004). Não houve diferenças entre PD-first, TR prévio e HD prévia em termos de óbito e sobrevida da técnica (p = 0,195 e p = 0,917, respectivamente) e a eficácia da DP foi adequada em todos os grupos.

**Conclusões:**

A DP é uma opção adequada para pacientes com DRET, independentemente da TRS anterior, e deve ser oferecida aos pacientes de acordo com seu status clínico e social e suas preferências.

## Introdução

A doença renal crônica (DRC) é, em grande parte, uma doença evitável e tratável que, segundo estimativas, afeta 9,1% da população mundial^1^. Uma taxa de filtração glomerular estimada (TFGe) <15 mL/min/1,73 m^2^ caracteriza a doença renal em estágio terminal (DRET)^2^, que pode ser tratada com diálise (hemodiálise ou diálise peritoneal), transplante renal ou uma abordagem conservadora.

Em 2010, 2.618 milhões de pessoas receberam terapia renal substitutiva (TRS) em todo o mundo. Entretanto, estima-se que entre 4.902 e 9.701 milhões de pessoas necessitem de TRS^3^. Disparidades na mortalidade associada à DRC revelam assimetrias regionais no acesso à diálise. Estima-se que 1 a 2 milhões de pessoas ao redor do mundo tenham ido a óbito prematuramente devido à falta de acesso à TRS em 2017^1,4^.

O transplante renal preemptivo (TR) com doador vivo é o tratamento de escolha para pacientes com DRC elegíveis para transplante^5^. Apesar dos muitos avanços na segurança e eficácia da imunossupressão que permitiram prolongar a sobrevida do enxerto, muitos pacientes precisam retornar à diálise após um período com um enxerto funcionante. De acordo com o *United States Renal Data System* (USRDS), a sobrevida ajustada do enxerto em 10 anos de doador vivo e doador falecido foi de 65,5% e 49,5%, respectivamente. Os pacientes que foram tratados com diálise peritoneal (DP) após a falha do enxerto tiveram maior probabilidade de receber um transplante renal subsequente do que morrer nos três anos seguintes; o oposto foi verdadeiro para os pacientes tratados com hemodiálise (HD)^6^.

Não existe uma modalidade preferida ao iniciar a diálise após uma falha no enxerto renal, uma vez que tanto a DP quanto a HD parecem ser opções adequadas^7–9^. O manejo ideal da imunossupressão em pacientes que iniciam a diálise com um TR malsucedido ainda é incerto, pois os dados nesse campo são escassos. Um enxerto malsucedido representa um estímulo inflamatório crônico que pode afetar negativamente o estado nutricional e o risco cardiovascular, e a preservação da função residual do enxerto pode impactar positivamente os desfechos da DP, como acontece com rins nativos. Os médicos devem avaliar os riscos e benefícios da suspensão da terapia imunossupressora. Em caso de manutenção da imunossupressão, os medicamentos antiproliferativos devem ser descontinuados primeiro. Os inibidores de calcineurina (ICN) devem ser reduzidos gradualmente ao longo de várias semanas e os glicocorticoides ao longo de vários meses, com o objetivo de preservar a função renal residual (FRR) e evitar a rejeição do enxerto renal^7,10^.

Embora onerosa^11^, a HD é atualmente a TRS mais comum oferecida a pacientes com DRET, apesar das recomendações atuais de que a HD deve ser a prioridade mais baixa em um programa de tratamento de DRC, após a prevenção da progressão da DRC, tratamento conservador quando apropriado, TR e DP^4,12^.

A associação da DP com melhores desfechos clínicos e relatados pelos pacientes é bem estabelecida. Esses benefícios incluem melhor preservação da FRR, melhor qualidade de vida, preservação de territórios vasculares para posterior construção de acesso vascular e melhores desfechos subsequentes de TR^13^. A DP possui diversas características que podem torná-la atraente como TRS preferencial tanto em países de baixa a média renda quanto em países de alta renda. É tecnicamente mais simples e apresenta melhor custo-benefício, requer uma proporção menor de enfermeiros por paciente, é mais viável em regiões rurais e remotas, proporciona maior equidade em ambientes com recursos limitados e pode melhorar a sobrevida nos primeiros anos^13^. Apesar de suas potenciais vantagens, somente 8–12% dos casos de DRET em todo o mundo estão sob DP^14,15^. Diversos fatores contribuem para as diferenças regionais nas modalidades de TRS, incluindo políticas e financiamentos governamentais para diálise, fatores do sistema e das instalações de saúde, comorbidades e adequação dos pacientes, bem como fatores da indústria^16^.

Pacientes em TRS crônica mudam frequentemente de modalidade devido a razões médicas ou sociais. *Transição* é o termo utilizado para o processo que deve incluir períodos de preparação e adaptação à nova realidade.

A ultrafiltração peritoneal e a capacidade difusiva geralmente diminuem com o tempo. Foi proposta uma definição unificada de falha da técnica de DP que inclui um desfecho composto de transferência para HD ou óbito^13^. A peritonite e as infecções relacionadas à DP são as principais causas de falha da técnica, o que está associado a uma maior mortalidade^13^. A FRR é um marcador substituto da DP fortemente associado a uma melhor sobrevida da técnica e do paciente^13^.

Inovações centradas no paciente que apoiem um tratamento renal mais eficiente e melhorem os desfechos dos pacientes são cada vez mais exigidas^17^. Neste estudo, nosso objetivo foi avaliar os desfechos de pacientes em DP de acordo com a modalidade de TRS anterior.

## Métodos

Este foi um estudo retrospectivo observacional unicêntrico aprovado pelo Comitê de Ética do nosso hospital. Avaliamos todos os pacientes com DRET que iniciaram DP em nossa unidade de 1º de janeiro de 2010 a 31 de dezembro de 2017. Os pacientes incidentes com menos de 3 meses sob DP foram excluídos. Os indivíduos foram acompanhados por 60 meses ou até a saída da DP. Foram incluídos pacientes em diálise peritoneal ambulatorial contínua (DPAC) e diálise peritoneal automatizada (DPA).

Selecionamos três coortes de pacientes. A coorte *PD-first* incluiu pacientes sem TRS prévia; o grupo HD prévia incluiu pacientes que fizeram a transição diretamente da HD; e o grupo TR prévio incluiu pacientes que fizeram a transição para DP diretamente de um TR malsucedido.

O índice de comorbidade de Charlson é uma ferramenta de prognóstico validada utilizada para avaliar a carga da doença e a taxa de mortalidade em 10 anos^18^. Calculamos a carga de comorbidade de acordo com o escore de Charlson.

O Teste de Equilíbrio Peritoneal (PET, por sua sigla em inglês) inicial foi realizado de 3–6 meses após o início da DP e, em seguida, a cada 6–12 meses, de acordo com as necessidades clínicas. Foi utilizado um protocolo modificado usando glicose a 3,86%/4,25%. As amostras de sangue foram coletadas no momento da infusão e 2 horas após o procedimento. As amostras de fluido peritoneal são coletadas em 0, 2 e 4 horas; o fluido peritoneal é completamente drenado 4 horas após a infusão. Foram analisados uma coleta de urina de 24 horas e um efluente peritoneal de 24 horas. Os resultados do PET incluíram Kt/V semanal, diurese e TFGe, razão D/P de creatinina e avaliação nutricional com a taxa catabólica de proteína normalizada (nPCR, por sua sigla em inglês)^19^. A capacidade de ultrafiltração (UF) também foi avaliada e a falência de UF foi definida como UF < 400 mL^20^.

A falha da técnica foi avaliada por meio de um desfecho composto de óbito ou transferência para HD^13^, e a sobrevida da técnica foi definida como o intervalo entre o início da DP e a falha da técnica. Em nosso centro, uma estratégia para reduzir a carga glicêmica em pacientes diabéticos foi implementada com o uso de soluções de diálise peritoneal sem glicose, como icodextrina ou soluções à base de aminoácidos^21^.

Os protocolos de imunossupressão para pacientes que iniciaram a DP a partir de um TR malsucedido incluíram a suspensão imediata de medicamentos antiproliferativos e a redução progressiva de ICN. Os glicocorticoides foram mantidos até que não houvesse diurese residual. Os pacientes que estavam anteriormente em uso de inibidores da proteína alvo da rapamicina em mamíferos (mTOR) mudaram para ICN antes da colocação do cateter de DP para evitar atrasos na cicatrização.

### Análise Estatística

As variáveis contínuas são apresentadas como média e desvio padrão para variáveis com distribuição normal ou como mediana e intervalo interquartil (IIQ) para variáveis com distribuição não normal, e as variáveis categóricas foram relatadas como frequências ou porcentagens. A análise estatística inferencial incluiu o teste de Kruskal-Wallis para comparar variáveis contínuas com distribuição não normal, ANOVA unidirecional para comparar variáveis contínuas com distribuição normal e teste qui-quadrado ou teste exato de Fisher para comparar variáveis categóricas.

Foram realizadas curvas de sobrevida de Kaplan-Meyer. A análise de regressão multivariada de Cox foi utilizada para analisar variáveis clínicas independentemente associadas à falha da DP (óbito ou transferência para HD) durante o período de acompanhamento. Variáveis com p < 0,20 ou selecionadas a critério do pesquisador na análise univariada foram incluídas no modelo multivariado. Foram consideradas significativas as diferenças com um valor de p < 0,05. A análise estatística foi realizada com o programa SPSS v. 22.0 (SPSS Inc., Chicago, IL, EUA).

## Resultados

Houve 156 pacientes que iniciaram a DP no período estudado, dos quais 4 foram excluídos devido ao tempo de acompanhamento insuficiente. Entre os 152 pacientes incluídos em nosso estudo, 115 foram PD-first, 22 fizeram transição da HD e 15 fizeram transição de um TR malsucedido (Figura 1). A mediana do tempo de acompanhamento foi de 45,72 meses (IIQ 37,44). A população incluiu 61,8% de pacientes do sexo masculino, com idade média de 51,1 ± 16,5 anos no início da DP. Mais de um terço (36,8%) tinha diabetes, e o escore mediano de Charlson foi de 4,0 (IIQ 4,0), com 51,3% dos pacientes apresentando um escore de 3–6. Quase um terço (29,6%) não apresentou comorbidades relevantes além de DRET (escore de Charlson de 2). Pacientes com escore de Charlson > 2 tiveram uma taxa mais elevada de falha da técnica (60,7% *vs.* 37,8%; p = 0,012). Não houve diferença estatística entre os grupos com relação à mortalidade geral e entre pacientes com escore de Charlson > 2 (Tabela 1).

**Figura 1:** Fluxograma de seleção e acompanhamento de pacientes. HD – Hemodiálise; TR – Transplante Renal; DP – Diálise Peritoneal.

**Tabela 1:** Dados demográficos, comorbidades e eventos clínicos dos pacientes estratificados por TSR anterior

Variável	Total	TRS anterior			p
		TR(n = 15)	HD(n = 22)	PD-first(n = 115)	
Idade no início da TRS (a), média ± DP	51,1 ± 16,5	30,0 ± 14,6	51,9 ± 13,5	53,8 ± 15,3	**<0,001**
Período em TRS (a), mediana (IIQ)	3,8 (3,1)	15,2 (8,0)	4,6 (2,8)	4,3 (2,7)	**<0,001**
Idade no início da DP (a), média ± DP	52,6 ± 15,2	41,0 ± 16,5	52,3 ± 15,4	53,0 ± 14,5	0,030
Tempo em DP (a), mediana (IIQ)	3,81 (3,12)	2,78 (3,20)	3,69 (3,19)	4,3 (2,6)	0,230
Sexo masculino, n (%)	94 (61,8)	6 (40,0)	12 (54,5)	76 (66,1)	0,110
Diabetes *mellitus*, n (%)	56 (36,8)	2 (13,3)	8 (36,4)	46 (40,0)	0,131
Hipertensão, n (%)	99 (65,1)	9 (60,0)	11 (50,0)	79 (68,7)	0,219
Escore de Charlson, mediana (IIQ)	4,0 (4,0)	2,0 (4,0)	4,0 (4,0)	4,0 (4,0)	0,151
Escore de Charlson, n (%)	–	–	–	–	0,231
2	45 (29,6)	8 (53,3)	5 (22,7)	32 (27,8)	–
3–6	78 (51,3)	6 (40,0)	14 (63,6)	58 (50,4)	–
Acima de 6	29 (19,1)	1 (6,7)	3 (13,6)	25 (21,7)	–
Taxa anual de internação hospitalar, mediana (IIQ)	0,60 (1,34)	1,08 (0,66)	0,38 (1,33)	0,40 (0,96)	**0,011**
Taxa anual de peritonite, mediana (IIQ)	0,21 (0,66)	0,70 (0,75)	0,10 (0,91)	0,21 (0,55)	0,065
IOS, n (%)	64 (42,1)	8 (53,3)	7 (31,8)	49 (42,6)	0,418
Infecção de túnel, n (%)	28 (18,4)	4 (26,7)	3 (13,6)	21 (18,3)	0,631
ECAM, n (%)	13 (8,6)	5 (33,3)	-	8 (7,0)	**0,004**
Saída temporária, n (%)	63 (41,4)	9 (60,0)	8 (36,4)	46 (40,0)	0,292
Saída, n (%)	–	–	–	–	0,494
Manutenção de DP	34 (22,4)	3 (20,0)	4 (18,2)	27 (23,5)	–
Transferência para hemodiálise	60 (39,5)	6 (40,0)	5 (22,7)	49 (42,6)	–
TR	33 (21,7)	4 (26,7)	7 (31,8)	22 (19,1)	–
Perda de acompanhamento	3 (2,0)	–	–	3 (2,6)	–
Óbito	22 (14,5)	2 (13,3)	6 (27,3)	14 (12,2)	0,195
Falha da técnica, n (%)	82 (53,9)	8 (53,3)	11 (50,0)	63 (54,8)	0,917

Sessenta e dois pacientes (40,8%) não apresentaram nenhum evento de peritonite durante o acompanhamento, 57,9% não tiveram infecção de sítio de saída e 81,6% não tiveram infecção de túnel. Não houve diferenças significativas entre os grupos na incidência de infecções de túnel e de sítio de saída, e observou-se uma tendência sem significância estatística para uma taxa anual de peritonite mais elevada no grupo TR prévio (mediana de 0,70 peritonite/ano por paciente *vs.* 0,10 em HD e 0,21 em DP; p = 0,065). Treze pacientes (8,6%) apresentaram um evento cardiovascular maior (ECAM), dos quais 5 haviam sido transferidos de um TR malsucedido (33,0% *vs.* 7,0% *vs.* 0%; p = 0,004). Aproximadamente um terço dos pacientes (n = 48, 31,6%) não teve nenhuma internação hospitalar durante o acompanhamento. No entanto, a taxa anual de internação hospitalar no grupo TR prévio foi de 1,08 internações/ano por paciente, superior à dos demais grupos (0,38 em HD e 0,40 em PD-first; p = 0,011). As principais razões para internação hospitalar no grupo TR prévio foram infecções relacionadas à DP (n = 19; 35,8%), eventos cardiovasculares (n = 13; 24,5%), infecções não relacionadas à DP (n = 6; 11,3%) e problemas não infecciosos relacionados à DP (n = 6; 11,3%). Não houve diferenças entre pacientes em DPA e DPAC com relação à taxa anual de admissão hospitalar ou à incidência de peritonite.

Os pacientes que fizeram a transição da HD para a DP apresentaram uma média de idade de 51,9 ± 13,5 anos e uma mediana de tempo em HD de 0,93 anos (IIQ 1,77). O principal motivo para a transição para DP foi a opção do paciente (n = 14; 63,6%), seguido por problemas com o acesso vascular para HD (n = 7; 31,8%) e intolerância à ultrafiltração na HD (n = 1; 4,5%).

Os pacientes no grupo TR prévio haviam recebido um enxerto renal com idade média de 33,0 ± 14,7 anos, e a duração do TR apresentou uma mediana de 9,8 anos (IIQ 5,0). Após a transição para DP, a imunossupressão sem glicocorticoides foi mantida por uma mediana de 181,5 dias (IIQ 176,0). Os glicocorticoides foram mantidos por um período mais longo, enquanto o paciente ainda apresentava FRR. Esse grupo era consideravelmente mais jovem quando iniciou a TRS (média de 30,0 *vs.* 51,9 em HD *vs.* 53,8 em PD-first; p < 0,001) e quando iniciou a DP (média de 41,0 *vs.* 52,3 em HD *vs.* 53,0 em PD-first; p = 0,030). Sua longevidade na DRET também é consideravelmente maior (mediana de 15,2 anos contra 4,6 em HD e 4,3 em PD-first; p < 0,001).

Dezesseis pacientes não realizaram nenhum PET, e 18 realizaram um primeiro PET, mas não realizaram um segundo exame devido à saída precoce da DP. A eficácia média da diálise foi adequada (Kt/V > 1,7) em todos os grupos, tanto no início da DP quanto no final do acompanhamento. A mediana da diurese no início da DP foi de 1.525 mL (IIQ 1.400), o que correspondeu a uma TFG mediana de 6,3 mL/min/1,73 m^2^ (IIQ 5,6). A taxa anual mediana de redução da diurese foi de 305 mL (IIQ 703).

Foram incluídos no estudo pacientes em DPAC (114) e DPA (38). Os pacientes sob DPAC eram mais velhos (54,9 *vs.* 46,1; p = 0,002). Não houve diferenças no Kt/V no início da DP (p = 0,591) ou no final do acompanhamento, bem como na TFG no final do acompanhamento. Pacientes sob DPAC apresentaram um índice de comorbidade mais elevado em comparação com pacientes sob DPA (índice de Charlson > 2 de 77,0% *vs.* 52,6%; p = 0,007).

No início da DP, havia 17 pacientes já anúricos. Três dos pacientes pertenciam ao grupo de TR prévio (20% de todos os TR prévios), 4 pertenciam ao grupo HD prévia (18,2% de todas as HD prévias) e 10 ao grupo PD-first (8,7% de todas as PD-first). Em relação à FRR, a diurese no início da DP foi menor no grupo TR prévio e maior no grupo PD-first (750 mL/dia *vs.* 1300 mL/dia *vs.* 1825 mL/dia; p < 0,001), assim como no final do acompanhamento (0 *vs.* 300 *vs.* 1250 mL; p < 0,001). A TFG seguiu o mesmo padrão (no início da DP 2,2 *vs.* 6,3 *vs.* 7,0 mL/min, p < 0,001; no final do acompanhamento 0,0 *vs.* 1,5 *vs.* 3,8 mL/min, p < 0,001). Os percentuais anuais de perda de diurese e TFG medida durante o acompanhamento foram maiores no grupo de pacientes que fizeram a transição de um TR malsucedido (percentual anual de perda de diurese de 0,59 em TR prévio *vs.* 0,13 *vs.* 0,13%, p = 0,044; e percentual anual de perda de TFG medida de 0,60 *vs.* 0,16 *vs.* 0,21%, p = 0,042).

Dezoito pacientes (11,8%) desenvolveram falência de UF durante o estudo. A falência de UF mostrou uma tendência – não significativa – de menor incidência no grupo TR prévio (6,7%) e maior no grupo HD prévia (27,3%; p = 0,070). Embora a maioria dos pacientes fossem médio-transportadores de soluto ao final do acompanhamento, 3,3% foram baixo-transportadores de soluto e uma porcentagem maior de pacientes (8,6%) foram alto-transportadores de soluto. O estado nutricional geral adequado também foi alcançado, embora tenha sido observada uma redução de uma nPCR inicial de 1,02 ± 0,28 para 0,89 ± 0,25. Não foi identificada diferença estatisticamente significativa entre os grupos (Tabela 2).

**Tabela 2:** Eficácia da diálise estratificada por TRS anterior

Variável	Total	TRS anterior			p
		TR(n = 15)	HD(n = 20)	PD-first(n = 101)	
D/P de creatinina no início da DP, média ± DP	0,69 ± 0,10	0,72 ± 0,03	0,67 ± 0,11	0,69 ± 0,11	0,492
D/P de creatinina no final do acompanhamento, média ± DP	0,68 ± 0,11	0,71 ± 0,10	0,66 ± 0,12	0,68 ± 0,10	0,487
Transporte peritoneal no final do acompanhamento	–	–	–	–	0,977
Baixo	5 (3,3)	–	1 (4,5)	4 (3,5)	–
Médio-baixo	43 (28,3)	4 (26,7)	7 (31,8)	32 (27,8)	–
Médio-alto	57 (37,5)	6 (40,0)	7 (31,8)	44 (38,3)	–
Alto	13 (8,6)	1 (6,7)	1 (4,5)	11 (9,6)	–
Kt/v (no início da DP), média ± DP	2,59 ± 0,81	2,24 ± 0,42	2,51 ± 0,60	2,69 ± 0,87	0,052
Kt/v (no final do acompanhamento), média ± DP	2,08 ± 0,62	1,79 ± 0,22	2,01 ± 0,76	2,12 ± 0,62	0,162
Taxa anual de alteração de Kt/v, média ± DP	0,25 ± 0,50	0,30 ± 0,36	0,17 ± 0,48	0,27 ± 0,52	0,716
Diurese no início da DP (mL), mediana (IIQ)	1525 (1400)	750 (1260)	1300 (1100)	1825 (1300)	**<0,001**
Diurese no final do acompanhamento (mL), mediana (IIQ)	1000 (1300)	0 (200)	300 (1465)	1250 (1175)	**<0,001**
Variação anual da diurese residual (%), média ± DP	0,17 ± 0,55	0,59 ± 0,41	0,13 ± 0,88	0,13 ± 0,49	**0,044**
TFG no início da DP (mL/min/1,73 m^2^), mediana (IIQ)	6,3 (5,6)	2,2 (5,1)	6,3 (7,5)	7,0 (5,4)	**<0,001**
TFG no final do acompanhamento (mL/min/1,73 m^2^), mediana (IIQ)	2,6 (4,7)	0,0 (0,6)	1,5 (3,6)	3,8 (4,5)	**<0,001**
Variação anual na TFG (%), média ± DP	0,24 ± 0,48	0,60 ± 0,39	0,16 ± 0,56	0,21 ± 0,47	**0,042**
Falência de UF, n (%)	18 (11,8)	1 (6,7)	6 (27,3)	11 (9,6)	0,070
nPCR no início da DP (g/kg/dia), média ± DP	1,02 ± 0,28	0,96 ± 0,17	0,95 ± 0,28	1,04 ± 0,28	0,198
nPCR no final do acompanhamento (g/kg/dia), média ± DP	0,89 ± 0,25	0,91 ± 0,19	0,81 ± 0,30	0,90 ± 0,25	0,382
Perda anual de nPCR (g/kg/dia), média ± DP	0,69 ± 0,21	0,02 ± 0,14	0,04 ± 0,14	0,08 ± 0,23	0,604

Sessenta e três pacientes (41,4%) estiveram temporariamente fora da DP devido a infecções ou cirurgias abdominais e retomaram a DP após um curto período. Por outro lado, a saída definitiva da DP antes dos 60 meses de acompanhamento ocorreu em 77,6% dos pacientes. Um total de 33 pacientes (21,7%) recebeu um TR durante o acompanhamento.

Metade de todos os pacientes (n = 82; 53,9%) desenvolveu falha da técnica (óbito ou transferência para HD) após uma mediana de 2,32 anos (IIQ 2,53) de DP. No geral, a maioria dos pacientes foi transferida para HD (39,5%) e os principais motivos foram falência de UF ou perda de eficácia da diálise (n = 21; 35%), complicações infecciosas relacionadas à DP (n = 20; 33%), problemas mecânicos relacionados ao cateter de DP (n = 7; 12%), perda de autonomia para realizar a DP e ausência de um auxiliar (n = 7; 12%) e opção do paciente (n = 5; 8%).

Apesar de uma taxa de mortalidade geral de 14,5%, houve uma tendência estatisticamente não significativa a uma taxa de mortalidade mais elevada em pacientes que fizeram a transição diretamente da HD (27,3% *vs.* 13,3% *vs.* 12,2%; p = 0,195). No grupo de pacientes que fizeram a transição diretamente da HD, os principais motivos para a saída da DP foram o TR (31,8%), seguido de óbito (27,3%).

Na análise multivariada, nem a saída da DP (p = 0,494) nem a sobrevida da técnica (p = 0,917; log rank = 0,612; Figura 2) foram diferentes entre os grupos. A taxa anual de internação hospitalar diminuiu tanto a sobrevida da técnica quanto a sobrevida do paciente (HR 1,536; IC95% 1,102–2,140; p = 0,011 e HR 1,797; IC95% 1,100–2,650; p = 0,017, respectivamente). O diabetes foi um fator de risco independente para óbito e para falha da técnica (HR 2,694; IC95% 1,102–6,586; p = 0,030 e HR 1,696; IC95% 1,047–2,747; p = 0,032) (Tabelas 3 e 4). Além disso, nem o tipo de DP (DPAC *vs.* DPA) nem o tipo de transição (PD-first *vs.* HD prévia *vs.* TR malsucedido) influenciaram a mortalidade geral ou a sobrevida da técnica.

**Figura 2.** Curvas de sobrevida da técnica de cada grupo para um desfecho composto de óbito ou transferência para hemodiálise, p = 0,612.

**Tabela 3:** Regressão de Cox para óbito

Variável	HR	IC 95%	p
Diabetes mellitus	2,694	1,102–6,586	0,030
Taxa anual de internação hospitalar	1,797	1,100–2,650	0,017

Variáveis no modelo: idade no início da DP, diabetes mellitus, internação hospitalar e taxa anual de peritonite.

**Tabela 4:** Regressão de Cox para falha da técnica

Variável	HR	IC 95%	p
Diabetes mellitus	1,696	1,047–2,747	0,032
Taxa anual de internação hospitalar	1,536	1,102–2,140	0,011

A falha da técnica é um desfecho composto de óbito e transferência para hemodiálise. Variáveis no modelo: débito urinário no início da DP, diabetes mellitus, internação hospitalar e taxa anual de peritonite.

## Discussão

A transição entre técnicas de TRS pode ser um evento estressante para os pacientes com DRET, que precisam adaptar-se a novos desafios e rotinas diárias. Nossa hipótese foi de que a técnica de TRS antes da DP poderia influenciar os desfechos da DP. Entre 152 pacientes, 115 eram PD-first, 22 fizeram a transição da HD e 15 vieram de um TR malsucedido. Os pacientes em transição de um TR malsucedido apresentaram tendência a uma maior taxa anual de peritonite e internações hospitalares. O débito urinário no final do acompanhamento foi menor em pacientes em transição de um TR malsucedido ou da HD. Pacientes em transição da HD apresentaram tendência a uma maior prevalência de falência de UF, o que não foi estatisticamente significativo. A presença de diabetes e internações hospitalares foram associadas a uma maior probabilidade de óbito ou transferência para HD.

A prevalência de diabetes entre os pacientes em DP foi de 36,8%, o que é menor do que na população geral em DP nos EUA (59,9%)^14^. Essas diferenças não parecem refletir as diferenças na prevalência de diabetes em ambos os países, que são semelhantes (cerca de 10%)^22,23^. Tanto a sobrevida do paciente quanto a da técnica foram influenciadas pela presença de diabetes. A probabilidade de óbito durante o acompanhamento mais do que dobrou em pacientes diabéticos (HR 2,694, IC95% 1,102–6,586; p = 0,030). O diabetes é um importante fator de risco cardiovascular e a doença cardiovascular é a principal causa de óbito em pacientes com DRET^24^. Embora os resultados não tenham mostrado significância estatística, os pacientes diabéticos tiveram uma incidência mais elevada de eventos cardiovasculares maiores durante o acompanhamento (14,3% *vs.* 5,2%; p = 0,072).

A probabilidade de falha da técnica – um desfecho composto de óbito e transferência para HD – foi 69,9% maior em pacientes diabéticos. Apesar da implementação de regimes de DP com redução de glicose em nossa unidade^21^, a exposição peritoneal à glicose em pacientes diabéticos em DP promove maior fibrose peritoneal, o que pode levar à falha da técnica. A sobrevida do paciente entre pacientes diabéticos em DP e HD é comparável. Cotovio et al.^25^ demonstraram que o diabetes foi um fator de risco independente para óbito, mas não para falha da técnica. Em sua análise, a taxa de peritonite foi semelhante entre pacientes não diabéticos e diabéticos, mas a taxa de hospitalização foi maior entre os diabéticos^25,26,27^.

O nível educacional parece estar associado ao risco de peritonite, independentemente do status econômico^28,29^. Em nossa população, a taxa anual geral de peritonite está abaixo das diretrizes recomendadas pela Sociedade Internacional de Diálise Peritoneal (ISPD) (0,21 *vs.* 0,40)^30^. No entanto, os pacientes que iniciaram a DP a partir de um TR malsucedido apresentaram uma taxa de peritonite mais elevada e esse foi o principal motivo de internação hospitalar nessa coorte. É interessante notar que a taxa de peritonite não influenciou a probabilidade de óbito ou falha da técnica. Por outro lado, a taxa anual de admissão hospitalar foi influenciada por ambas as variáveis. A probabilidade de óbito aumentou em 79,7% e a falha da técnica aumentou em 53,6% para cada ponto de aumento na taxa anual de internações. A taxa de admissão hospitalar reflete as complicações da DP e da DRET, como eventos infecciosos e cardiovasculares, que têm impacto nos desfechos da DP.

Em geral, a eficácia da diálise (Kt/V semanal) foi adequada, independentemente da TRS anterior, estado de diabetes ou modalidade de DP (DPA *vs.* DPAC). Todos os grupos perderam progressivamente a diurese e a função renal residual. No entanto, os pacientes com PD-first apresentaram um volume urinário diário significativamente maior do que outros grupos ao final do acompanhamento. A perda de volume urinário no grupo TR prévio pode refletir a perda acelerada da função renal residual no TR com a retirada progressiva da imunossupressão e a possível rejeição crônica do enxerto. Os pacientes que fizeram a transição da HD também desenvolveram menor débito urinário no final do acompanhamento. A DP parece preservar a função renal residual melhor do que a HD^31,32,33^.

Um problema adicional foi observado entre os pacientes que fizeram a transição da HD – uma tendência à maior incidência de falência de UF. Não há uma explicação clara para esse achado inesperado. Esse foi o grupo com a menor incidência de infecções – um fator de risco importante para a insuficiência de UF. A incidência de diabetes não foi maior do que a do grupo PD-first, que apresenta uma taxa de falência de UF muito menor. O grupo TR prévio – que teve pelo menos uma cirurgia abdominal importante e a menor incidência de diabetes, apesar dos anos de imunossupressão – apresentou a menor incidência de insuficiência de UF. Novamente, podemos especular se a estimulação inflamatória desencadeada pelo circuito extracorpóreo pode influenciar tanto a função renal residual quanto a função da membrana peritoneal. A tendência a uma maior prevalência de falência de UF e menor débito urinário em pacientes em transição da HD pode sugerir que o controle do estado de volume nesse grupo é mais desafiador.

Nossos achados foram comparados com uma coorte espanhola (n = 906) de pacientes em DP que não tinham TRS anterior ou que fizeram a transição de um TR malsucedido. Descobrimos que nossa coorte era mais jovem (51,1 *vs.* 54,8; p = 0,008) e apresentou um índice de comorbidade mais baixo (4,0 *vs.* 5,1; p < 0,001). Nossa coorte apresentou maior incidência de diabetes (36,8% *vs.* 24,0%; p < 0,001), maior mortalidade (14,5 *vs.* 9,7%; p = 0,084) e maior taxa de transferência para HD (39,5% *vs.* 17,0%; p < 0,001)^8^. Os melhores desfechos na coorte espanhola podem estar associados a uma prevalência muito menor de diabetes.

Os pacientes em HD prévia estiveram em HD por um período mediano de menos de um ano antes da transferência para DP. O primeiro motivo para a saída da DP foi o TR (31,8%) – mais do que qualquer outro grupo. Isso pode refletir o fenômeno de “transição repentina” na HD antes de decidir sobre a preferência pela DP como ponte para o TR. Curiosamente, os pacientes que iniciaram a DP a partir da HD também foram o grupo com a maior taxa de mortalidade. Conforme discutido anteriormente, a tendência de maior taxa de falência de UF e menor débito urinário no final do acompanhamento pode ter afetado os desfechos.

Durante os 8 anos de acompanhamento, apenas 9,9% dos pacientes que iniciaram a DP eram provenientes de TR. O grupo TR prévio apresentou uma taxa anual de peritonite significativamente maior (0,70 episódios/ano por paciente) em comparação com outros pacientes em DP, o que está acima do recomendado pela ISPD^30^. Eventos cardiovasculares maiores também foram mais comuns nesse grupo (33,0% *vs.* 7,0% *vs.* 0; p = 0,004). Provavelmente, a perda acelerada de diurese associada à uremia de longa duração e à imunossupressão crônica favorece o desenvolvimento de doenças cardiovasculares e infecções. No entanto, não foram encontradas diferenças na eficácia da diálise, e os pacientes com TR prévio apresentaram as mesmas taxas de falha da técnica e óbito que a população em geral. Portanto, apesar do maior risco de infecção e de doenças cardiovasculares, a DP continua sendo uma opção adequada para pacientes com TR malsucedido. O controle dos fatores de risco cardiovascular deve ser intensificado com mais orientações sobre modificações no estilo de vida, como abandono do tabagismo, controle do peso corporal, adoção de uma dieta saudável e exercícios^34^. Fatores de risco cardiovascular, como diabetes e hipertensão, mas também doenças mineral-ósseas, devem ser controlados cuidadosamente. A intensificação de sessões de aprendizagem com enfermeiros especializados deve ser oferecida regularmente a esses pacientes, a fim de minimizar erros técnicos que possam levar a infecções^30,35^.

Nosso estudo é limitado por ser unicêntrico, retrospectivo e observacional, e apresentou assimetria no tamanho dos grupos estudados. Particularmente, houve um número menor de pacientes nos grupos TR e HD prévios. Além disso, o número limitado de pacientes que fizeram a transição de um TR malsucedido não permitiu a análise de diferentes estratégias de suspensão da imunossupressão após o início da DP. No entanto, esse estudo incluiu todos os pacientes de uma unidade sem pré-seleção e teve um longo acompanhamento. Até onde temos conhecimento, este é um dos primeiros estudos que comparou simultaneamente três coortes de pacientes em DP de acordo com sua TRS anterior, ou seja, HD *vs.* TR *vs.* PD-first.

## Conclusões

Avaliamos uma grande população de pacientes prevalentes em DP de acordo com sua TRS anterior com um longo acompanhamento. Os pacientes em transição da HD ou de um TR prévio parecem ter menor débito urinário ao final do acompanhamento. Uma forte tendência a uma taxa mais elevada de falência de UF em pacientes em transição da HD pode antecipar dificuldades no controle do estado do volume nesse grupo. Os pacientes que fizeram a transição a partir de um TR malsucedido apresentaram uma taxa maior de peritonite e internações hospitalares. A presença de diabetes e admissão hospitalar foram associadas a uma maior probabilidade de óbito ou transferência para HD. Os indicadores de eficácia da DP foram adequados em todos os grupos estudados.

Apesar das diferenças descritas anteriormente e de acordo com a literatura, a DP parece ser uma escolha válida de TRS crônica após TR ou HD malsucedidos e deve ser oferecida aos pacientes de acordo com seu status clínico e social e suas preferências.
